# Association of Glycated Hemoglobin Levels with Amputation Outcomes in Patients with Diabetic Foot Ulcers: A Cross-Sectional Study in Southern Iran (2023–2024): Effect of HbA1c on Diabetic Foot Ulcer Prognosis

**DOI:** 10.31661/gmj.v14i.3967

**Published:** 2025-08-29

**Authors:** Sahar Heidarykhayat, Kazem Jamali, Hamid Zaferani Arani, Zahra Abbasy, Mohammad Hadi Niakan

**Affiliations:** ^1^ Department of Surgery, Shiraz University of Medical Sciences, Shiraz, Iran; ^2^ Trauma Research Center, Shahid Rajaee (Emtiaz) Trauma Hospital, Shiraz University of Medical Sciences, Fars Province, Shiraz, Iran; ^3^ Department of Pediatrics, Tehran University of Medical Sciences, Tehran, Iran

**Keywords:** Diabetes Mellitus Type 2, Diabetic Foot, Hemoglobin A Glycosylated, Amputation

## Abstract

**Background:**

Diabetic foot ulcer (DFU) is the most serious consequence of type 2 diabetes mellitus (T2DM), frequently resulting in lower limb amputations. Glycated hemoglobin (HbA1c) acts as a marker of long-term glycemic control, but its prognostic value in predicting the severity and level of amputation in DFU patients remains undefined. This study aims to investigate the association between HbA1c levels and lower limb amputation risk at various anatomical levels in patients with DFU.

**Materials and Methods:**

This cross-sectional analytical study included 446 patients with T2DM and DFU admitted to Namazi Hospital, Shiraz, Iran, between February 2023 and January 2024. Patients were categorized into four groups based on the amputation level: above-knee, below-knee, foot/midfoot/forefoot, and toe/finger. HbA1c was measured and categorized into three groups (7–7.9%, 8–8.9%, and ≥9%). Univariate and multinomial logistic regression analyses were performed using the above-knee amputation group as the reference. Statistical analysis was conducted in R (v4.4.1), with significance set at P0.05.

**Results:**

Elevated HbA1c levels were significantly associated with increased odds of below-knee and foot amputations compared to above-knee amputation (OR=6.27, P=0.023 and OR=8.16, P=0.036, respectively, for HbA1c 8–8.9%). Similar associations were observed for HbA1c ≥9%. Additionally, patients who underwent less extensive amputations (i.e., foot and finger) tended to be younger and had shorter hospital stays compared to those who underwent above-knee amputations. Lower triglyceride levels were also associated with finger amputation (OR=0.99, P=0.031). These comparisons were made within a population in which all patients had diabetic foot ulcers.

**Conclusion:**

Higher HbA1c levels are significantly associated with more distal amputation levels in patients with DFU. Patient characteristics such as younger age, shorter hospitalization, and lower triglyceride levels were more common among those undergoing less extensive amputations. These findings highlight the need for early glycemic control and careful clinical monitoring in DFU patients at risk of major limb loss.

## Introduction

Diabetes mellitus (DM) is a lifelong metabolic disease that is most frequently type 2 diabetes (T2DM), which can be found in 90% to 95% of cases [1]. Diabetic foot ulcers (DFUs) are one of the most unpleasant and hardest to treat complications that affect the quality of patients' lives and the healthcare system [2]. DFUs have a significant impact on the lives of patients and are responsible for high rates of amputation of the lower limbs [3]. Even though diabetes care has developed, DFUs still pose a serious challenge, and thus, new ways to predict and prevent this situation are called for [4].

DFUs and other diabetes-related complications have been associated with hemoglobin A1c (HbA1c), a biomarker and a risk factor for long-term glycemic management [5]. Increased HbA1c levels are associated with macro/microvascular disorders and signify inadequate glycemic control [6].

However, little is known about the association between HbA1c levels and the prognosis of DFU, particularly in different populations.

The multifactorial pathophysiology of DFUs involves peripheral neuropathy, ischemia, and impaired wound healing consequent to chronic hyperglycemia. These factors interact in a complicated interplay that aggravates the risk of infection and delays healing [7]. While HbA1c supplies a glycemic measurement, its role in indicating the severity and healing process of DFUs is not fully apprehended. Moreover, identifying certain HbA1c thresholds that might direct clinical decision-making and ameliorate complications should be the subject of ongoing investigation [8].

Wrapping these gaps facilitates the development of specialized interventions and improves the management of DFUs in T2DM patients.

The incidence of diabetes and DFUs has been steadily increasing in Iran, reflecting global trends [9]. Despite the progress in diagnostic and therapeutic modalities, DFUs remain a momentous public health concern due to delayed diagnosis, inadequate glycemic control, and limited access to targeted therapy [10]. This emphasizes the demand for region-specific studies to sufficiently understand the predictors of DFU consequences and invent tailored approaches for prevention and management. HbA1c, as a readily available and cost-effective biomarker, is ensuring to response these challenges in resource-limited settings.This study aims to investigate the association between HbA1c levels and the prognosis of DFUs in T2DM patients admitted to Namazi Hospital in Shiraz, Iran. Identifying precise HbA1c thresholds relative to DFU outcomes, this research seeks to provide evidence-based insights, report clinical practice and improve patient care.

## Materials and Methods

### Study Design and Setting

This cross-sectional analytical study was conducted at Namazi Hospital, a referral center in Shiraz, Iran, from February 2023 to January 2024. This study followed the Declaration of Helsinki and national ethical guidelines for medical research. Participants were informed about the study objectives, procedures, and potential risks, and written informed consent was obtained. Patient data was maintained anonymized to keep confidentiality, and access to the dataset was confined to authorized personnel only. The study was approved by the Research Ethics Committee (REC) of Shiraz University of Medical Sciences (Approv-al ID: IR.SUMS.MED.REC.1403.688).

### Study Population and Sampling

The study population comprised adult patients (aged ≥18 years) diagnosed with T2DM and presenting with DFUs. Patients were excluded if they had type 1 diabetes, gestational diabetes, chronic kidney disease (stage ≥3), malignancies, or other clinically significant systemic conditions known to affect wound healing or inflammatory status, such as advanced liver disease or autoimmune disorders. The sample size was estimated using the standard formula for calculating a single population proportions: n=(Z² × P × (1 - P)) / d², where Z is the Z-score for a 95% confidence level (1.96), P is the estimated prevalence of elevated HbA1c in DFU patients (0.7931), and d is the desired margin of error (0.04) [11][10].

### Data Collection

Data were collected using clinical examination and a structured questionnaire, including demographic information (age, gender, education level, and occupation), clinical history (duration of diabetes, history of DFUs, and comorbidities), and lifestyle factors (smoking and physical activity). Clinical data contained HbA1c levels, fasting blood sugar (FBS), erythrocyte sedimentation rate (ESR), white blood cell (WBC) count, and lipid profile components including triglycerides and cholesterol. DFU severity was categorized by the Wagner classification system, which divides ulcers into six grades 0 through 5 based on the depth and extent of tissue involvement. For the purpose of analysis, ulcer severity was dichotomized into Wagner grade 4 (deep ulcer with osteomyelitis or abscess) and grade 5 (extensive gangrene or necrosis) [12]. All tests performed using standardized methods at the hospital's central laboratory.

### HbA1c Measurement and Categorization

HbA1c levels were measured using high-performance liquid chromatography (HPLC), a gold-standard method for glycated hemoglobin inspection [13]. Patients were divided into three groups based on their HbA1c levels: 7-7.9%, 8-8.9%, and ≥9%. These thresholds were chosen based on previous investigations that determined significant differences in DFU outcomes across these categories [14][15][16]. The primary variable of interest was the association between HbA1c levels and DFU prognosis, including healing duration, infection rates, and the risk of amputation (if any) during the index hospitalization.

### Outcome Measures

The primary outcomes of the study were the occurrence and anatomical level of lower extremity amputation in patients with diabetic foot ulcers. Amputations were categorized into four clinically distinct groups: above-knee amputation (AKA), below-knee amputation (BKA), foot/midfoot/forefoot amputation, and toe or finger amputation. Each category represented a separate outcome and was analyzed independently as a binary variable to identify specific clinical and biochemical predictors associated with the likelihood of undergoing that particular level of amputation. This approach allowed for a nuanced assessment of how different factors contribute to varying severities of limb loss.

### Statistical Analysis

All statistical analyses were conducted using R software (version 4.4.1, IBM Training - United States). Due to the presence of overlapping ulcer locations in individual patients, and in order to ensure the mutual exclusivity of outcome categories, participants with multiple concurrent ulcer sites were excluded from the analysis. After this data refinement step, the final analytical sample included 305 unique individuals.

Continuous variables were summarized as medians with interquartile ranges (IQRs) due to their non-normal distributions, while categorical variables were reported as frequencies and percentages. In the univariate analysis phase, differences in continuous variables across ulcer location groups were assessed using the Kruskal-Wallis test, and differences in categorical variables were evaluated using the chi-square test. Candidate variables for the adjusted multinomial logistic regression model were selected based on a univariate screening threshold of P<0.2 and/or established relevance in the literature. A multinomial logistic regression model was then fitted to identify independent predictors of ulcer location, using above-knee amputation as the reference group. All statistical tests were two-sided, and a P-value less than 0.05 was considered statistically significant.

## Results

**Table:** Table1. Demographic and Clinical Data of T2DM Patients with DFU

Variable	Total
Number of patients	446
Age (years)	66.1 ± 14.2
Triglyceride (mg/dL)	187.3 ± 53.9
ESR (mm/hr)	60.5 ± 27.3
Diabetes duration (years)	21.1 ± 8.1
Duration of diabetic wound infection (months)	2.7 ± 2.6
Duration of hospitalization (days)	8.8 ± 4.4
Wagner grade 4	325 (72.9)
Wagner grade 5	121 (27.1)
HbA1c 7-7.9% (%)	47 (10.5)
HbA1c 8-8.9% (%)	134 (30.0)
HbA1c ≥ 9% (%)	265 (59.4)
Female gender (%)	166 (37.2)
Male gender (%)	280 (62.8)
Smoking (Yes) (%)	283 (63.5)
Smoking (No) (%)	163 (36.5)
Education (Below Diploma) (%)	271 (61.0)
Education (Above Diploma) (%)	173 (39.0)

**Note:** Continuous variables are presented as Mean±SD ; categorical variables are reported as frequency (percentage).

**Table:** Table2. Univariate Analysis of Baseline Demographic and Clinical Characteristics Stratified by Amputation Category

**Predictor**	AKA	BKA	Finger Amputation	Foot Amputation	P-value
Age (years)	73.5 (64 - 80)	64.03(56 - 73)	61(54 - 70.25)	67.5(54.75 - 75)	**0.009**
Hospital stays (days)	12(8.5 - 14)	8(7 - 12)	5(4 - 6)	7(5 - 8)	**0.001**
Triglyceride level	219(154 - 250.5)	184(154 - 228.5)	161(1336.5-186.75)	175.5(153-195.75)	**0.0001**
Diabetes duration (years)	20(17.25 - 30)	20(15 - 24.5)	17(12 - 24)	19(15 - 27)	**0.027**
ESR (mm/hr)	69(53 - 84)	67(46.25 - 81)	62.5(22 - 78)	66(29 - 81)	0.252
Duration of diabetic wound infection (months)	3.79 (2.00 - 4.20)	2.96 (2.00 - 3.00)	2.43 (2.00 - 3.00)	2.31 (2.00 - 3.19)	**0.040**
HbA1c (7-7.9)	7(20.6%)	11(9.6%)	15(14.4%)	4(7.7%)	0.127
HbA1c (8-8.9)	9(26.5%)	33(28.7%)	38(36.5%)	18(34.6%)	
HbA1c (≥9)	18(52.9%)	71(61.7%)	51(49.0%)	30(57.7%)	
Wagner grade 4	26(76.5%)	88(76.5%)	94(90.4%)	44(84.6%)	**0.038**
Wagner grade 5	8(23.5%)	27(23.5%)	10(9.6%)	8(15.4%)	
Smoking (No)	11(32.4%)	38(33.0%)	49(47.1%)	23(67.6%)	0.125
Smoking (Yes)	23(67.6%)	77(67.0%)	55(52.9%)	29(55.8%)	
Female gender	25(73.5%)	73(63.5%)	58(55.8%)	32(61.5%)	0.301
Male gender	9(26.5%)	42(36.5%)	46(44.2%)	20(38.5%)	
Job (Unemployed)	16 (47.1%)	45 (39.1%)	39 (37.5%)	25 (48.1%)	0.254
Job (Self-employed)	11 (32.4%)	46 (40.0%)	36 (34.6%)	12 (23.1%)	
Job (Employee)	7 (20.6 %)	21 (18.3%)	29 (27.9%)	15 (28.8%)	
Job (Student)	0	3 (2.6%)	0	0	
Education (Below Diploma)	27 (79.4%)	71 (61.7%)	43 (41.7 %)	27 (52.9%)	**0.0005**
Education (Above Diploma)	7 (20.6%)	44 (38.3%)	60 (58.3%)	24 (47.1%)	

**Note:**Continuous variables are presented as median (interquartile range); categorical variables are reported as frequency (percentage). Variables with P<0.2 were included in the multivariable model. Bolded P-values indicate statistical significance at P<0.05.

**Table:** Table3. Adjusted Odds Ratios (ORs) and 95% Confidence Intervals (CIs) for Predictors of Ulcer Location from the Multinomial Logistic Regression Model (reference category: above-knee)

**Predictor**	**Outcome Group **	**OR**	**95% CI (Lower-Upper) **	**P-value**
HbA1c (8-8.9%)	BKA	6.27	1.32 - 29.89	**0.023**
HbA1c (≥9%)	BKA	5.59	1.48 - 21.14	**0.011**
Age (years)	BKA	0.95	0.90 - 1.00	0.072
Hospital stays (days)	BKA	0.92	0.82 - 1.03	0.156
Triglyceride level	BKA	1.00	0.99 - 1.01	0.499
Wagner grade 5 (%)	BKA	1.72	0.46 - 6.41	0.422
Smoking (Yes) (%)	BKA	1.4	0.49 - 3.96	0.529
Education (Above Diploma) (%)	BKA	0.86	0.15 - 4.99	0.866
HbA1c (8-8.9%)	Finger Amputation	5.89	0.91 - 38.1	0.062
HbA1c (≥9%)	Finger Amputation	4.73	0.90 - 24.94	0.067
Age (years)	Finger Amputation	0.96	0.9 - 1.02	0.211
Hospital stays (days)	Finger Amputation	0.37	0.29 - 0.49	**0.001**
Triglyceride level	Finger Amputation	0.99	0.98 - 1.00	**0.031**
Wagner grade 5 (%)	Finger Amputation	2.66	0.52 - 13.64	0.242
Smoking (Yes) (%)	Finger Amputation	0.81	0.24 - 2.72	0.737
Education (Above Diploma) (%)	Finger Amputation	1.67	0.25 - 11.33	0.599
HbA1c (8-8.9%)	Foot Amputation	8.16	1.14 - 58.56	**0.036**
HbA1c (≥9%)	Foot Amputation	5.92	1.00 - 35.01	**0.048**
Age (years)	Foot Amputation	0.96	0.90 - 1.02	0.172
Hospital stays (days)	Foot Amputation	0.68	0.56 - 0.81	**0.001**
Triglyceride level	Foot Amputation	0.99	0.98 - 1.00	0.201
Wagner grade 5 (%)	Foot Amputation	1.61	0.35 - 7.49	0.544
Smoking (Yes) (%)	Foot Amputation	0.91	0.28 - 2.96	0.875
Education (Above Diploma) (%)	Foot Amputation	1.1	0.16 - 7.49	0.92

**Note:**Estimates are adjusted odds ratios (ORs) with 95% confidence intervals (CIs), obtained from a multinomial logistic regression model using “above-knee” as the reference group. The model was adjusted for potential confounders including sex, duration of diabetes and Duration of diabetic wound infection. Bolded values indicate statistically significant associations at P<0.05. Abbreviations: BKA, below-knee amputation; ORs, odds ratios; CIs, confidence intervals.

**Figure-1:**
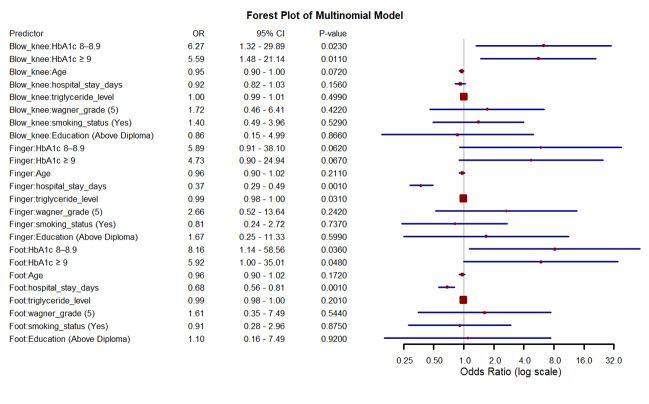
Figure 1. Adjusted odds ratios with 95% confidence intervals for predictors of ulcer anatomical location from the multinomial logistic regression model (reference category: above-knee). Abbreviations: ORs, odds ratios; CIs, confidence intervals.

### Demographic and Clinical Data of Patients

A total of 446 patients with DFUs , the mean age of participants was 66.1 ± 14.2 years, and 62.8% were male. The average duration of diabetes was 21.1 ± 8.1 years, with a mean diabetic wound infection duration of 2.7 ± 2.6 months. The average hospital stay was 8.8 ± 4.4 days. Laboratory findings revealed a mean triglyceride level of 187.3 ± 53.9 mg/dL and an ESR of 60.5 ± 27.3 mm/hr. According to the Wagner classification, 72.9% of patients were categorized as grade 4 and 27.1% as grade 5, revealing advanced ulcer severity. Remarkably, 59.4% of patients had HbA1c levels ≥ 9%, indicating poor glycemic control (Table-1).

### Association of Clinical Variables with Amputation Types

Table-2 presents the results of the univariate analysis comparing patient demographic and clinical characteristics across four anatomical ulcer location groups: above-knee amputation (AKA), below-knee amputation (BKA), foot/midfoot/forefoot, and finger amputation. Significant differences (P<0.05) were observed across groups for age, hospital stay duration, triglyceride levels, diabetes duration, duration of diabetic wound infection, education and Wagner grade, indicating their potential association with ulcer location. Variables with P-values less than 0.2 were included in the subsequent multinomial logistic regression.

Table-3 presents the final results of the multinomial logistic regression model assessing the association between selected patient characteristics and the anatomical location of diabetic foot ulcers, using the above-knee amputation (AKA) group as the reference category. The model was adjusted for sex, diabetes duration, and wound infection duration. In the below-knee amputation (BKA) group, higher HbA1c levels were significantly associated with greater odds of amputation compared to the AKA group. Patients with HbA1c levels of 8-8.9% had over six times the odds of undergoing BKA (OR=6.27, P=0.023), while those with HbA1c ≥9% also had significantly higher odds (OR=5.59, P=0.011) relative to the reference category. A similar association was observed in the foot amputation group, where elevated HbA1c levels were also significantly associated with increased odds of foot amputation compared to AKA (OR=8.16, P=0.036 for HbA1c 8-8.9%; OR=5.92, P=0.048 for HbA1c≥9%). In this group, shorter hospital stays were also significantly associated with foot amputation compared to AKA (OR=0.68, P=0.001). This indicates that for each additional day of hospitalization, the odds of foot amputation as opposed to above-knee amputation decreased by 32%. Although age did not reach statistical significance in any group, the direction of association was consistent. For example, in the BKA group, each one-year increase in age was associated with a 5% decrease in the odds of BKA compared to AKA (OR=0.95; 95% CI: 0.90-1.00). This indicates that younger patients were more likely to undergo less extensive amputations, while older patients were more often classified in the AKA group. In the finger amputation group, two additional variables were significantly associated with ulcer location. Shorter hospital stays were observed in this group compared to AKA (OR=0.37, P<0.001). Additionally, lower triglyceride levels were associated with finger amputation in comparison to AKA (OR=0.99, P=0.031). This means that with each unit increase in triglyceride level, the odds of finger amputation as opposed to above-knee amputation decreased by approximately 1%. While the effect size is small, the association was statistically significant. This forest plot displays the adjusted ORs and 95% CIs for the association between HbA1c levels and other clinical predictors with the anatomical location of diabetic foot ulcers. Estimates were derived from a multinomial logistic regression model, using "above-knee" ulcers as the reference category. Each horizontal line represents the 95% CI for the corresponding OR, and the central point on each line indicates the point estimate (OR). The vertical dashed line at OR=1 denotes the null value (no association). ORs greater than 1 suggest increased odds of ulcer occurrence in the given location compared to the reference, while ORs less than 1 indicate decreased odds. (Figure-1).

## Discussion

DFU is a severe and debilitating complication of T2DM, often culminating in lower limb amputation. The intricate interplay between chronic hyperglycemia, peripheral neuropathy, ischemia, and immune dysfunction impairs wound healing and increases infection risk, making DFUs difficult to manage and predict in clinical trials [1][2]. HbA1c, a well-established marker of long-term glycemic control, has been widely associated with the development of diabetic complications, including microvascular and macrovascular pathologies [3][4].

However, the role of HbA1c as a prognostic indicator for DFU consequences—particularly in predicting amputation risk at different anatomical levels—has been incompletely apprehended. This study strived to elucidate this relationship by analyzing the association between HbA1c levels and amputation severity among hospitalized T2DM patients with DFU at Namazi Hospital, Iran. The findings provide insight into the differential predictive value of glycemic control regarding limb preservation, offering important implications for individualized clinical decision-making. In this cohort of 446 patients with DFUs, the mean age was 66.1 ± 14.2 years, with a predominance of male patients (62.8%) and a mean diabetes duration of 21.1 ± 8.1 years. These findings are compatible with previous reports, which indicate that DFUs most generally occur in elders with long-standing diabetes, mainly among men with higher risk due to delayed care-seeking ways and higher rates of peripheral arterial disease [17][18][19]. A systematic review by Li et al. (2025) conveyed a pooled mean age of 63.1 years for patients with DFUs, with males including nearly 60% of the population, supporting the demographic trends of our study [20].

The high prevalence of smoking in our sample (63.5%) is also notable, as smoking is a known risk factor for impaired wound healing and increased risk of lower limb amputation in diabetic patients [3].

Regarding ulcer severity, most of our patients showed advanced lesions since 72.9% of them were categorized as Wagner grade 4 and 27.1% as grade 5. This distribution reflects the results of Yazdanpanah et al. (2024), who, in a regional Iranian cohort, also observed a high incidence of late-stage DFUs, typically ascribed to delayed referrals and restricted access to specialized wound care centers [9]. Regarding ulcer severity, most of our patients showed advanced lesions since 72.9% of them were categorized as Wagner grade 4 and 27.1% as grade 5. This distribution reflects the results of Bikramjit et al. (2017 , who, in a regional Iranian cohort, also observed a high incidence of late-stage DFUs, typically ascribed to delayed referrals and restricted access to specialized wound care centers [11].

Our analysis revealed a distinct and level-specific association between long-term glycemic control and the risk of lower limb amputation. Elevated HbA1c levels (≥9%) were intensely associated in the case of below-knee and foot/midfoot/forefoot amputations with significantly increased odds of undergoing amputation at these levels. Patients with HbA1c ≥9% had significantly higher probabilities of undergoing below-knee amputation (OR=5.35; P=0.015) and foot/midfoot/forefoot amputation (OR=5.94; P=0.048), for example, than those with HbA1c between 7-7.9%. These results compromise the several studies linking poor glycemic control to worse DFU consequences, including longer wound healing times and raised amputation rates.

Akyüz et al. (2023) underscored hyperglycemia's negative impact on wound healing and infection control by finding that HbA1c levels above 9% were significantly associated with both surgical extension and ulcer severity [14].

According to Tong et al. (2022), higher HbA1c levels were also associated with delayed healing and altered local skin microbiota, so possibly exacerbating ulcer risk and infection [16].

Interestingly, higher HbA1c levels (≥8%) were significantly associated with increased odds of distal amputations—specifically below-knee and foot-level procedures—compared to above-knee amputation, which served as the reference category in the multinomial regression model.

Contrary to earlier assumptions and some prior reports, our analysis did not reveal a protective or inverse relationship between elevated HbA1c levels and above-knee amputation. Instead, patients with HbA1c levels of 8-8.9% and ≥9% were more likely to undergo amputations at lower anatomical sites, while no statistically significant association was identified for the likelihood of undergoing above-knee amputation in relation to HbA1c levels.

This finding partially contrasts with earlier studies such as that by Wan et al. (2023) and Klein & Buse (2020), which proposed a potential inverse association in critically ill patients due to stress-induced hypoglycemia or suppressed HbA1c levels in catabolic states like sepsis or malnutrition [15][21].

However, in our sample, which comprised mostly patients with chronic, advanced DFUs rather than acute systemic deterioration, these confounding factors may have had limited influence. Additionally, Akyüz et al. (2023) and Tong et al. (2022) highlighted strong positive associations between high HbA1c and poor healing, infection severity, and need for surgical intervention—findings that align well with the current study’s emphasis on HbA1c as a predictor of more frequent distal amputations, though not necessarily of more extensive ones such as above-knee procedures [14][16]. Therefore, our results suggest that elevated HbA1c may play a more prominent role in the pathophysiological processes leading to limb loss at lower levels, likely through mechanisms involving delayed wound healing, neuropathy, and peripheral ischemia, rather than being a straightforward predictor of proximal amputation.

Altogether, HbA1c remains a valuable prognostic biomarker in predicting the risk of distal amputations. Our findings indicate that elevated HbA1c [22] (particularly ≥8%) is significantly associated with increased odds of below-knee and foot-level amputations, while its role in predicting higher-level (above-knee) amputations remains undefined due to lack of direct comparison in the statistical model. Thus, the interpretation of HbA1c should be contextualized based on clinical presentation and anatomical outcomes.

## Conclusion

This research underscores the level-specific association between glycated hemoglobin and lower extremity amputation in patients with diabetic foot ulcers. Poor glycemic control, as reflected by elevated HbA1c levels (≥9%), was significantly associated with an increased risk of more distal amputations, including below-knee and foot-level procedures. Contrarily, higher HbA1c levels arose to be inversely associated with above-knee amputation, suggesting that such proximal outcomes may be influenced by additional clinical or systemic factors beyond long-term glycemic status. Other factors such as age, triglyceride levels, and length of hospitalization were also found to be independently associated with amputation risk. These findings support the use of HbA1c as a context-dependent risk indicator and underscore the need for individualized assessment strategies in diabetic foot management. Also, longitudinal studies are attested to explore the reason and potential mechanisms underlying these associations and to specify tailored HbA1c thresholds for conducting clinical decision-making in diverse diabetic populations.

## Conflict of Interest

The authors declare that they have no conflict of interest with respect to the author or publication of this article.
